# Serum Biomarkers as Predictors of Recurrence in Chronic Rhinosinusitis with Nasal Polyps

**DOI:** 10.3390/medicina62081452

**Published:** 2026-07-27

**Authors:** Maria-Delia Florean, Codruța Mare, Anda Gâta, Veronica Elena Trombitaș, Liviuța Budișan, Oana Zanoaga, Ioana Berindan-Neagoe, Carmen Gabriela Stelea, Alexandra Roman, Silviu Albu

**Affiliations:** 1 Department of Otorhinolaryngology and Head and Neck Surgery, “Iuliu Hatieganu” University of Medicine and Pharmacy, 400349 Cluj-Napoca, Romania; florean_mariadelia@yahoo.com (M.-D.F.); silviualbu63@gmail.com (S.A.); 2 Statistics, Forecasting, Mathematics Department, Faculty of Economics and Business Administration, Babeş-Bolyai University, 400591 Cluj-Napoca, Romania; 3 MedFuture Biomedical Research Institute, “Iuliu Hatieganu” University of Medicine and Pharmacy, 400349 Cluj-Napoca, Romania; lbudisan@yahoo.com (L.B.);; 4 Department of Oral Surgery, Faculty of Dental Medicine, “Grigore T. Popa” University of Medicine and Pharmacy, 700115 Iasi, Romania; 5 Department of Periodontology, Faculty of Dental Medicine, “Iuliu Hatieganu” University of Medicine and Pharmacy, 400012 Cluj-Napoca, Romania

**Keywords:** eosinophilia, eotaxin, procalcitonin, periostin, IL-33, relapse

## Abstract

*Background and Objectives*: Chronic rhinosinusitis with nasal polyps (CRSwNP) is a heterogeneous type 2 inflammatory disease characterized by frequent postoperative recurrence despite endoscopic sinus surgery (ESS). Reliable biomarkers capable of predicting recurrence remain insufficiently validated. This study evaluates the prognostic utility of circulating biomarkers for recurrence prediction in CRSwNP, in addition to the predictive performance of the blood eosinophilia. *Materials and Methods*: A prospective cohort of 69 patients with CRSwNP undergoing primary ESS was followed for up to 3 years. Periostin, eotaxin-3, procalcitonin and IL-33 were taken from blood before surgery and analyzed by ELISA technique. Clinical outcomes were assessed using SNOT-22 and Perioperative Sinus Endoscopy (POSE) scores. Statistical analyses included Spearman’s correlation, the Mann–Whitney U test, the Kruskal–Wallis test, Cox proportional hazards regression, multivariable binary logistic regression, ROC analysis, and longitudinal MANCOVA. *Results*: Blood eosinophilia remained the strongest clinical predictor of recurrence (OR = 4.55, *p* = 0.001). Serum periostin, eotaxin and procalcitonin significantly correlated with postoperative disease severity and recurrence, particularly with POSE scores at 1 and 3 years (*p* < 0.05). Patients with recurrence demonstrated significantly higher serum levels of these biomarkers compared with controlled patients (all *p* < 0.001). Multivariate Cox regression identified periostin (HR = 1.033, *p* = 0.010), eotaxin (HR = 1.554, *p* < 0.001) and procalcitonin (HR = 1.183, *p* < 0.001) as significant predictors of recurrence. Procalcitonin demonstrated the highest predictive performance in ROC analysis (AUC = 0.805). Longitudinal MANCOVA confirmed significant associations between periostin, eotaxin, procalcitonin, and persistent postoperative inflammatory burden over 3 years. *Conclusions*: Circulating periostin, eotaxin and procalcitonin are promising predictors of CRSwNP recurrence and appear to contribute to prognostic accuracy. Integration of serum biomarker profiling into clinical practice may improve personalized postoperative management and risk stratification in CRSwNP.

## 1. Introduction

Chronic rhinosinusitis (CRS) is an inflammatory disease with high prevalence, affecting approximately 11% of the European population [1]. According to the 2020 European Position Paper on Rhinosinusitis and Nasal Polyps (EPOS), CRS is primarily categorized based on clinical phenotype into CRS with nasal polyps (CRSwNP) and CRS without nasal polyps (CRSsNP). However, this anatomical classification alone does not fully explain the marked clinical heterogeneity of CRS. Increasing attention has therefore shifted toward endotypic classification in type 2 and non-type 2 inflammation, which characterizes the underlying immunopathological mechanisms and supports the implementation of personalized therapeutic strategies [2].

Chronic rhinosinusitis is currently recognized as a heterogeneous inflammatory disorder resulting from complex interactions between host immune responses, epithelial barrier dysfunction, genetic susceptibility, and environmental exposures. Among these mechanisms, allergic (atopic) inflammation contributes to disease development in a subset of patients, although its role varies considerably across different CRS phenotypes and endotypes. Recent evidence has identified Central Compartment Atopic Disease (CCAD) as a pathophysiologically distinct subtype of CRS strongly associated with inhalant allergy, further emphasizing the heterogeneity of inflammatory pathways underlying chronic sinonasal disease [3]. CCAD further illustrates how integrating anatomical features with underlying immunological mechanisms may improve disease phenotyping and facilitate more individualized diagnostic and therapeutic approaches, supporting the transition toward precision medicine in chronic rhinosinusitis. Recent advances on European Academy of Allergy and Clinical Immunology Position Paper (EAACI) position paper in the understanding of hypersensitivity reactions recognize that immune-mediated diseases may involve both IgE-mediated and non-IgE-mediated mechanisms. Accordingly, the inflammatory endotypes observed in CRSwNP likely reflect complex cellular- and cytokine-driven immune responses involving coordinated innate and adaptive pathways, emphasizing the multifactorial immunobiology of the disease and supporting endotype-based patient stratification [4].

Within the spectrum of CRSwNP, type 2 inflammation represents the predominant inflammatory endotype and is associated with T helper 2 (Th2)-mediated immune responses. This subgroup can be further stratified into eosinophilic (eCRSwNP) and non-eosinophilic (neCRSwNP) forms, depending on the degree of eosinophilic infiltration within the sinonasal mucosa [5]. These endotypes are not only immunologically distinct but also clinically relevant, as eosinophilic CRSwNP is consistently linked to increased disease severity, reduced responsiveness to conventional therapies, and a higher likelihood of postoperative recurrence [6].

Periostin (POSTN), an extracellular matrix protein induced by IL-4 and IL-13, has emerged as a key mediator linking inflammation to tissue remodeling and promotes eosinophilic infiltration and fibrosis [7]. Notably, EPOS 2020 identifies periostin as a relevant biomarker for the characterization of type 2 endotype in bilateral CRSwNP [2]. Procalcitonin (PCT),a prohormone precursor of calcitonin that is expressed primarily in parafollicular C cells of the thyroid gland and lung has recently emerged as a potential mediator of chronic airway inflammation. Experimental evidence suggests that its expression is regulated by pro-inflammatory cytokines, including TNF-α, IL-1β, and IL-6, and increased serum and tissue PCT levels have been demonstrated in patients with CRSwNP, supporting its potential involvement in persistent mucosal inflammation and tissue remodeling [8].

IL-33 is secreted by various types of immune cells, such as macrophages and dendritic cells, and is constitutively expressed in epithelial tissues and lymphoid organs [9]. Studies demonstrated that IL-33 is a chemoattractant for Th2 cells [10] and stimulates the eosinophils, mast cells, basophils, and natural killer cells, promoting the release of proinflammatory cytokines IL-5 and IL-13 [11] and contributing to the pathogenesis of chronic respiratory diseases [12]. Eotaxins represent a family of potent eosinophil-specific chemoattractants that regulate leukocyte trafficking to inflamed sinonasal tissue via endothelial adhesion mechanisms [13]. Among them, eotaxin-3 has been reported to correlate with tissue eosinophilia, and elevated plasma levels may serve as a predictive marker for disease recurrence [14].

Despite substantial advances in surgical management, CRSwNP remains characterized by a high recurrence rate following endoscopic sinus surgery (ESS), with more than half of patients experiencing relapse within three years [15]. Early identification of patients at increased risk of recurrence would facilitate individualized postoperative surveillance and improve long-term disease management. Therefore, the present study aimed to investigate the prognostic value of selected serum biomarkers in predicting postoperative recurrence of CRSwNP and to evaluate their potential role in preoperative risk stratification within a personalized medicine framework.

## 2. Materials and Methods

### 2.1. Study Design, Setting and Participants

This study was conducted in accordance with the Declaration of Helsinki, and the research protocol was approved by the Institutional Ethics Committee of the “Iuliu Hațieganu” University of Medicine and Pharmacy, Cluj-Napoca, Romania (Approval Nos. 590/date: 10 December 2019 and 23/date: 20 February 2024). Written informed consent was obtained from all participants prior to inclusion in the study. Patients were recruited from the Department of Otolaryngology of CF Cluj Clinical Hospital, Cluj-Napoca, Romania, between March 2020 and November 2021 and followed postoperatory until 2025. The study cohort included 69 patients diagnosed with CRSwNP who underwent primary ESS.

All patients underwent comprehensive clinical evaluation, including physical examination, nasal endoscopy, and computed tomography (CT). The diagnosis of CRSwNP was established according to the criteria outlined in the European Position Paper on Rhinosinusitis and Nasal Polyps (EPOS 2020) [2]. Inclusion criteria were patients with CRSwNP undergoing primary surgery, who failed maximal medical therapy. Patients undergoing revision surgery were excluded. Additional exclusion criteria included: age under 18 years, autoimmune diseases, immunodeficiency, primary ciliary dyskinesia, fungal rhinosinusitis, inverted papilloma, antrochoanal polyps, sinonasal malignancies, and the use of antibiotics or other immunomodulatory therapies within 4 weeks prior to surgery.

Preoperative variables included demographic data (age, sex) and clinical characteristics such as smoking status, environmental allergies, nonsteroidal anti-inflammatory drug (NSAID) hypersensitivity, and comorbid bronchial asthma. Subjective assessment included patient-reported outcome measures: the Sino-Nasal Outcome Test-22 (SNOT-22) and the Patient Health Questionnaire-9 (PHQ-9). Objective assessment included nasal endoscopy scores, CT imaging scores, and peripheral blood eosinophil counts. CT findings were evaluated using the Lund–Mackay scoring system [16], while endoscopic findings were graded according to the Lildholdt score [17].

### 2.2. Sample Collection and Biomarker Analysis

Peripheral venous blood samples (5 mL) and polyp tissue were collected from each participant prior to ESS. Samples were allowed to clot at room temperature for 1–2 h and subsequently centrifuged at 1200 rpm for 10 min at 4 °C. Serum was separated and stored at −80 °C until further analysis. Serum levels of periostin (POSTN), eotaxin-3 (CCL26), procalcitonin (PCT), IL-33 were quantified using enzyme-linked immunosorbent assay (ELISA) kits, according to the manufacturers’ instructions.

Protein quantification was performed using commercially available sandwich ELISA kits: POSTN (Elabscience, Houston, TX, USA; Cat. No. E-OSEL-H0013), CCL26 (R&D Systems, Minneapolis, MN, USA; Cat. No. DCC260B), PCT (Elabscience, Houston, TX, USA; Cat. No. E-EL-H1492), IL33 (BioLegend/8999 BioLegend Way/San Diego, CA 92121 U.S.A., Cat.no.435907). Absorbance was measured using a BioTek Synergy H1 microplate reader (BioTek Instruments, Inc., Winooski, VT, USA for POSTN, CCL26, and IL-33) and a SpectraMax 190 microplate reader (Molecular Devices, LLC, San Jose, CA, USA for PCT). Concentrations were calculated based on standard curves using GraphPad Prism 6 software.

Nasal polyp tissue samples collected during ESS were used for miRNA analysis. Total RNA was extracted, and the expression of miR-125b and miR-203a-3p was quantified by quantitative real-time PCR (RT-qPCR), as previously described [18]. These analyses were performed to explore the relationship between circulating serum biomarkers and tissue molecular markers associated with CRSwNP pathogenesis.

### 2.3. Follow-Up and Recurrence Evaluation

Patients were monitored postoperatively at 6 months, 1 year and 3 years. At each follow-up visit, nasal endoscopy was performed, and patients completed the Sino-Nasal Outcome Test-22 (SNOT-22), representing subjective outcome measures. Objective evaluation was performed using the Perioperative Sinus Endoscopy (POSE) scoring system after Wright and Agrawal [19], carried out by the same examiner. POSE score was the variable for disease status classiffcation into three categories: controlled (POSE ≤ 4), partially controlled (POSE 5–7), and relapse (POSE ≥ 8) [20]. For recurrence prediction analyses (logistic regression, Cox proportional hazards regression, and ROC analysis), patients with controlled and partially controlled disease were analyzed as a single non-recurrence group, while recurrence was defined as a POSE score ≥ 8.

### 2.4. Statistical Analysis

The distribution of continuous variables was assessed using visual inspection of histograms, Q–Q plots and detrended Q–Q plots. These were finally validated using both the Shapiro–Wilk and the Kolmogorov–Smirnoff normality tests. Skewness, kurtosis, and homogeneity of variances (Levene’s test, where appropriate) were also evaluated. Continuous variables are presented as the mean ± standard deviation (SD) or median (interquartile range, IQR), according to data distribution, whereas categorical variables are presented as frequencies and percentages. Normally distributed variables (age, preoperative SNOT-22, and serum procalcitonin) were analyzed using the independent-samples *t*-test, while non-normally distributed variables were compared using the Mann–Whitney U test. Categorical variables were compared using Pearson’s χ^2^ test. Correlations were assessed using Spearman’s rank correlation coefficient, and comparisons among more than two groups using the Kruskal–Wallis test followed by Mann–Whitney U post hoc tests.

Peripheral blood eosinophilia was evaluated as an established clinical predictor of postoperative recurrence. Its discriminative performance was assessed using receiver operating characteristic (ROC) analysis, and multivariable binary logistic regression was performed to evaluate its association with recurrence. In parallel, serum periostin, eotaxin, procalcitonin, and IL-33 were investigated as circulating biomarkers with potential prognostic value.

Serum biomarkers were initially evaluated using univariate analysis, followed by multivariate analysis and Cox proportional hazards regression. Variables included in the multivariable logistic and Cox regression models were selected based on their biological relevance and statistically significant associations identified in the univariate analyses. For the Cox proportional hazards analysis, time-to-event was defined as the interval between endoscopic sinus surgery and the first endoscopic evidence of postoperative recurrence (POSE score ≥ 8). Longitudinal changes and recurrence patterns were assessed using repeated-measures multivariate analysis of covariance (MANCOVA), considering follow-up time points and combined dependent variables, while adjusting for relevant covariates. The dependent variables were POSE scores assessed at 6 months, 1 year and 3 years, while Total Relapse was entered as a fixed factor and each biomarker as a continuous covariate. Multivariate significance was evaluated using Wilks’ Lambda and Pillai’s Trace, with *p* < 0.05 considered statistically significant. Logistic regression models were evaluated using ROC curve analysis. The area under the curve (AUC), as well as sensitivity and specificity, were calculated to assess predictive performance. All statistical analyses were performed using SPSS software (version 31; IBM Corp., Chicago, IL, USA). During the preparation of this manuscript, the authors used Generative Artificial Intelligence (GPT-5.5) for language editing and improvement of English expression. The authors have reviewed and edited the output and take full responsibility for the content of this publication.

## 3. Results

A total of 69 patients with CRSwNP were enrolled in this prospective study. Baseline characteristics of patients according to recurrence status are presented in Table 1. No significant differences were observed between groups regarding age, sex, smoking status, NSAID intolerance, preoperative SNOT-22 score, Lund–McKay score, or endoscopic score (all *p* > 0.05).

In contrast, asthma (85.0% vs. 16.3%, *p* < 0.001) was significantly more frequent among patients with recurrence. All patients with relapse presented high levels of blood eosinophilia (100% vs. 16.3%, *p* < 0.001) and multivariable logistic regression demonstrated a significant association between peripheral blood eosinophilia and postoperative recurrence (OR = 4.55, 95% CI: 1.86–11.15; *p* = 0.001).

Patients who developed recurrence had significantly higher pre-operatory blood periostin, eotaxin and procalcitonin levels. Serum IL-33 levels did not differ significantly between the two groups.

Given the strong predictive value of peripheral eosinophilia, circulating biomarkers were subsequently evaluated to determine whether they could provide additional prognostic information for postoperative recurrence.

Baseline serum periostin, eotaxin, and procalcitonin levels demonstrated significant positive correlations with preoperative radiological and endoscopic disease severity. Eotaxin showed the strongest association with the Lund–Mackay score, while procalcitonin exhibited the strongest correlation with the preoperative endoscopic score. In contrast, none of the investigated biomarkers were significantly associated with baseline SNOT-22 scores. Serum IL-33 did not demonstrate significant correlations with any of the preoperative disease severity measures (Table 2).

Spearman’s correlation analysis revealed significant associations between seric biomarker levels and clinical and radiological severity scores. Significant correlations were observed with postoperative Lund–Mackay scores, with the strongest association identified for procalcitonin, followed by eotaxin and periostin. Regarding patient-reported outcomes, eotaxin demonstrated the strongest correlations with SNOT-22 scores at both 1 year and 3 years. Similar but weaker associations were observed for periostin and procalcitonin. Stronger correlations were identified between these biomarkers and POSE scores. Eotaxin exhibited a very strong association with POSE scores at 1 year, which remained significant at 3 years. Comparable associations were observed for periostin and procalcitonin. Serum IL-33 wasn’t corelated with Lund–Mackay, SNOT-22, neither POSE any time (Table 3).

Periostin, eotaxin, and procalcitonin showed significant positive correlations with tissue miR-125b expression (*p* > 0.05), whereas no significant correlations were observed for serum IL-33. In contrast, miR-203a-3p was not significantly associated with any of the investigated circulating biomarkers (*p* > 0.05) (Table 4).

**Figure 1:**
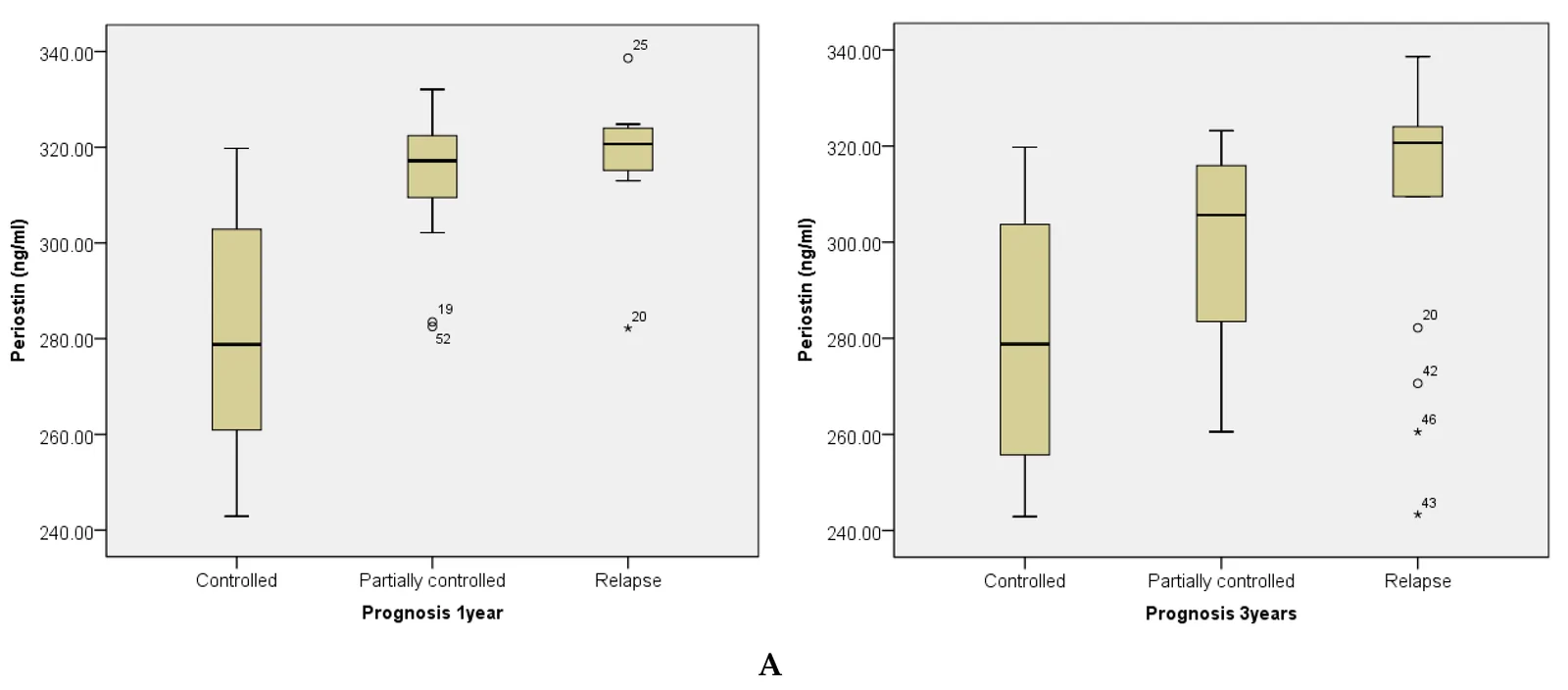

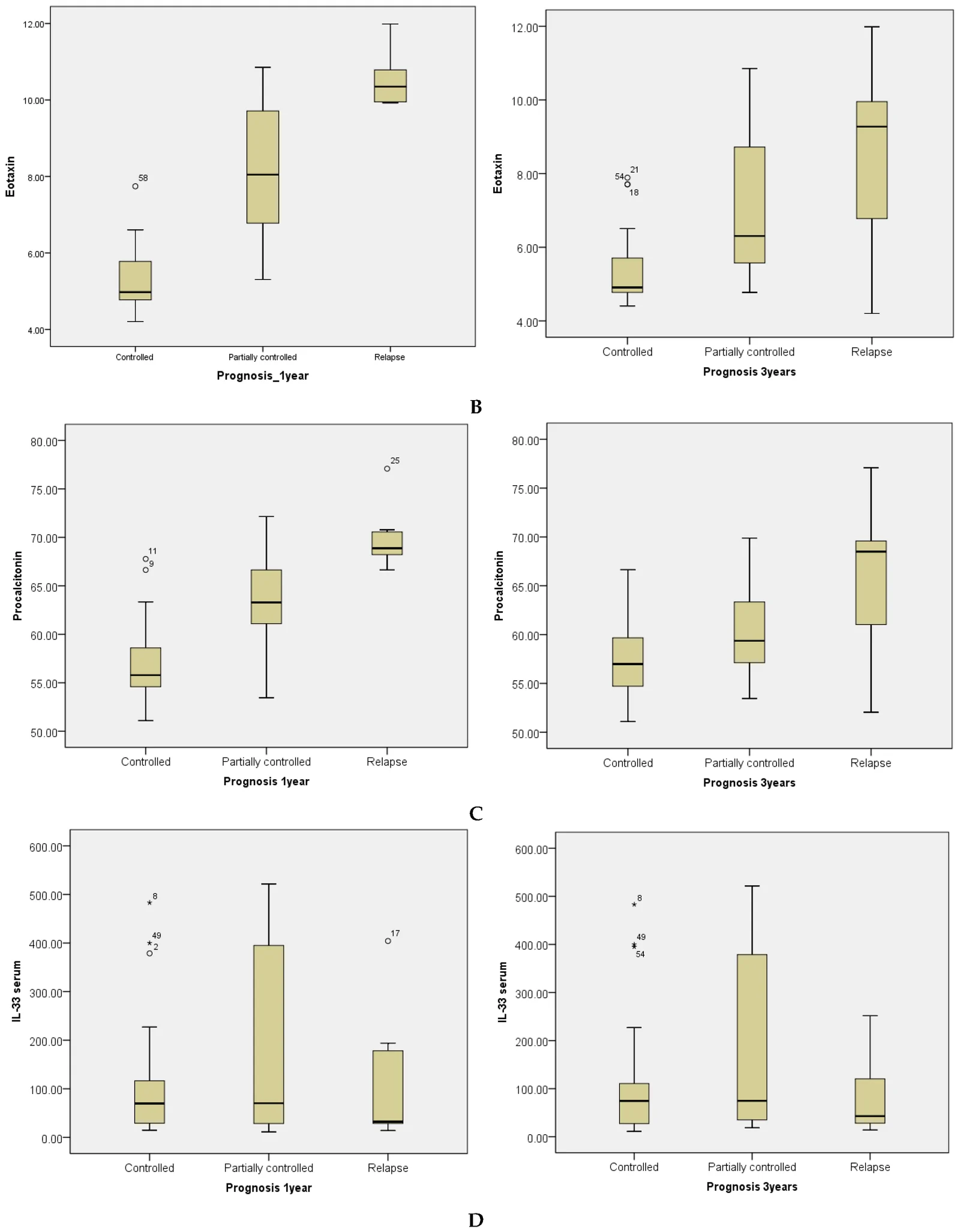
Boxplot distribution of periostin (**A**), eotaxin (**B**), procalcitonin (**C**), and serum IL-33 (**D**) levels according to 1-year and 3-year prognosis groups (controlled, partially controlled, and relapse), according to recurrence status.

Univariate non-parametric analysis was performed using the Mann–Whitney U test to evaluate the association between biomarker levels and total relapse. Serum periostin, eotaxin, and procalcitonin levels were significantly higher in patients with recurrence compared to those without recurrence. In contrast, serum IL-33 levels did not differ significantly between the two groups (Table 5 and Figure 1).

A multivariate Cox proportional hazards regression model was performed to identify predictors of nasal polyposis recurrence during follow-up. Variables included in the multivariate model were periostin, eotaxin, serum IL-33, procalcitonin, with hazard ratio and statistical *p*-value presented in Table 6.

**Figure 2:**
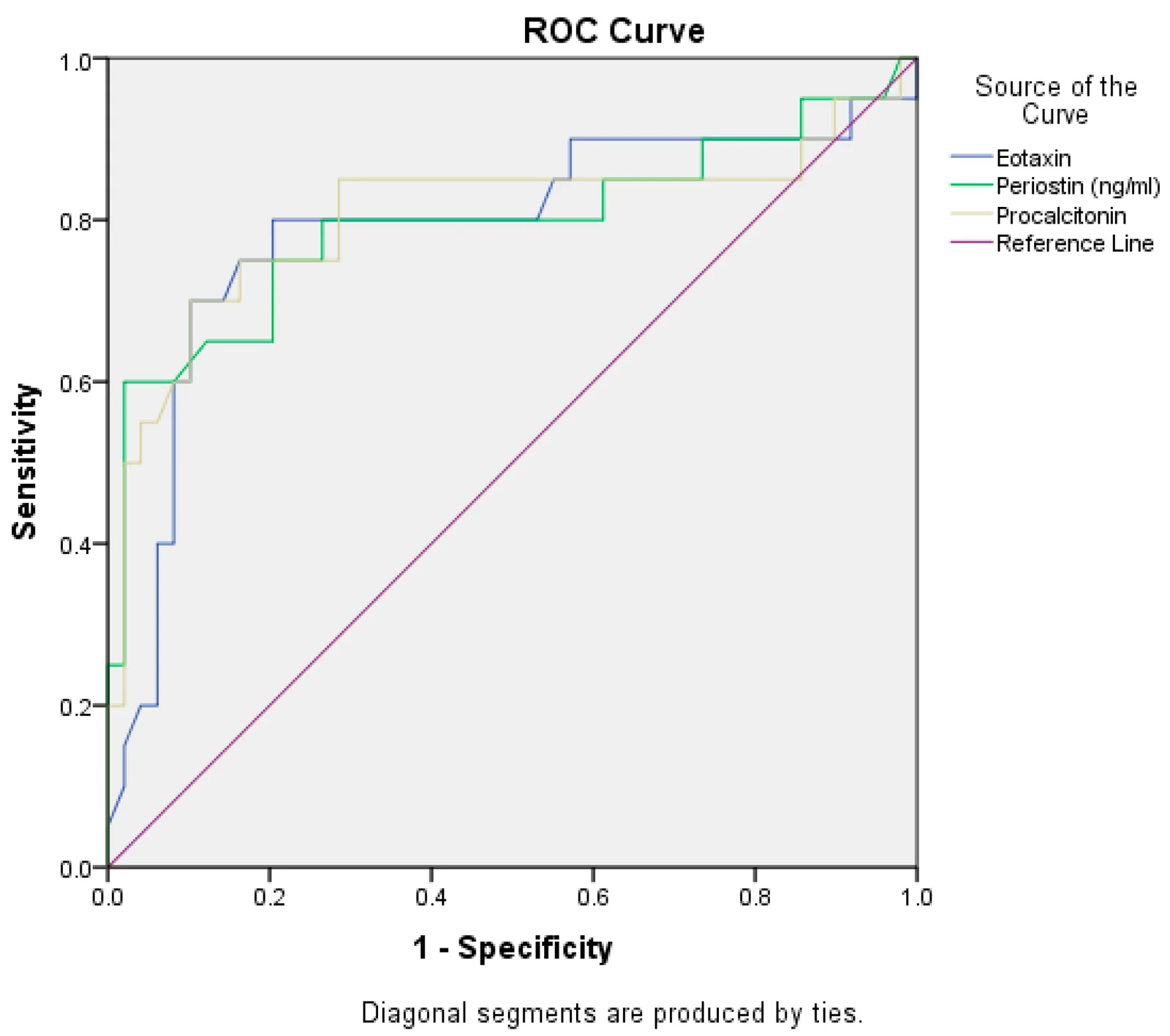
Time-dependent ROC curve analysis showing the performance of the model in predicting disease recurrence at 3 years. The curves illustrate the sensitivity–specificity trade-off across thresholds, while the diagonal line represents chance-level discrimination.

ROC curve analysis showed that peripheral blood eosinophilia had the highest predictive performance among the evaluated clinical predictors. Among the investigated circulating biomarkers, procalcitonin demonstrated the highest discriminative ability, followed by periostin and eotaxin. In contrast, serum IL-33 did not significantly predict recurrence, as reflected by the non-significant *p*-values and lower AUC values (Table 7). Overall, the predictive performance of procalcitonin, periostin and eotaxin decreased over time but remained clinically relevant at 3 years (Figure 2).

Kruskal–Wallis analysis revealed significant differences in periostin, eotaxin and procalcitonin levels across prognosis categories at 3 years (periostin: χ^2^ = 16.621, *p* < 0.001; eotaxin: χ^2^ = 20.701, *p* < 0.001; procalcitonin: χ^2^ = 16.376, *p* < 0.001).

**Figure 3:**
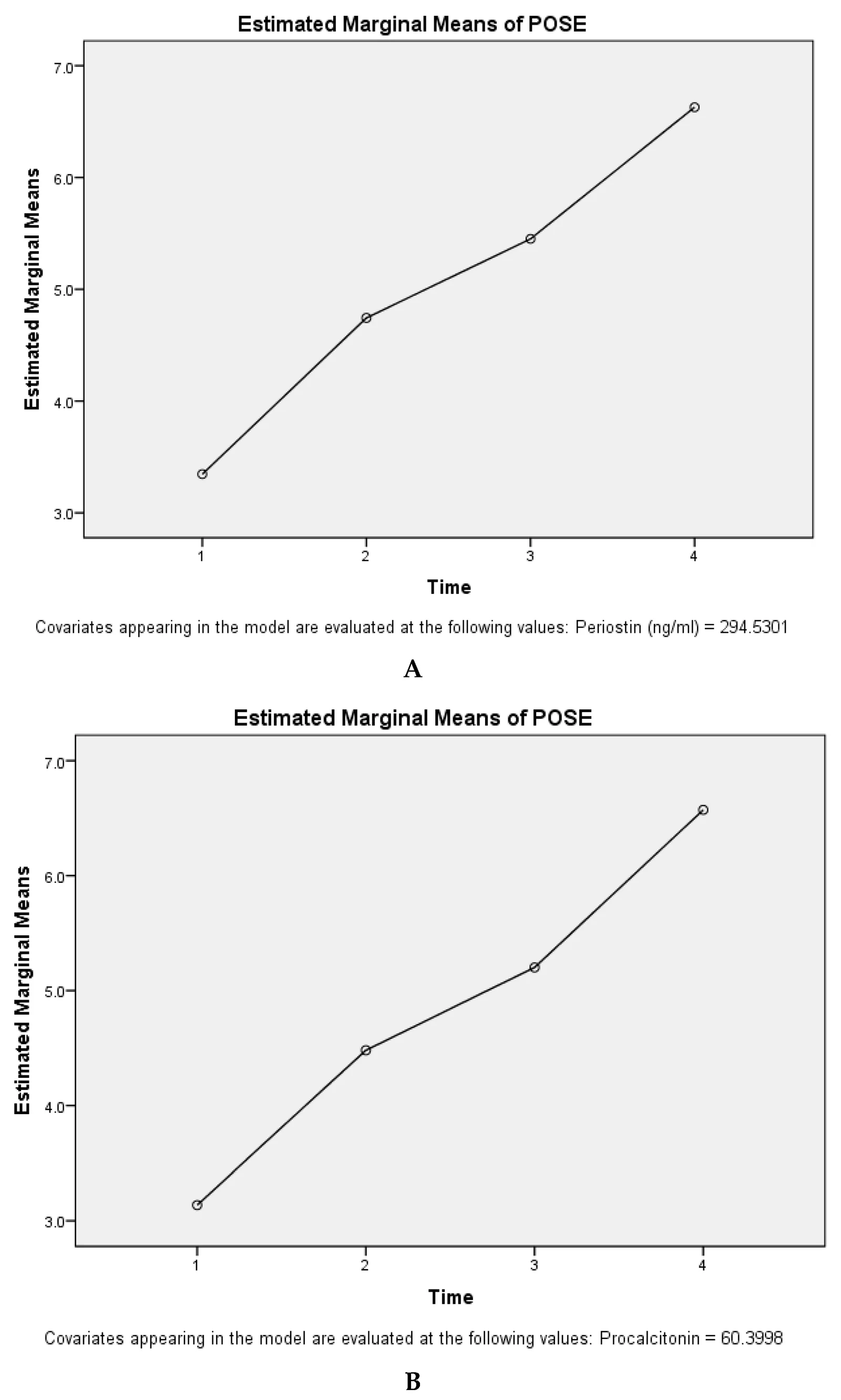

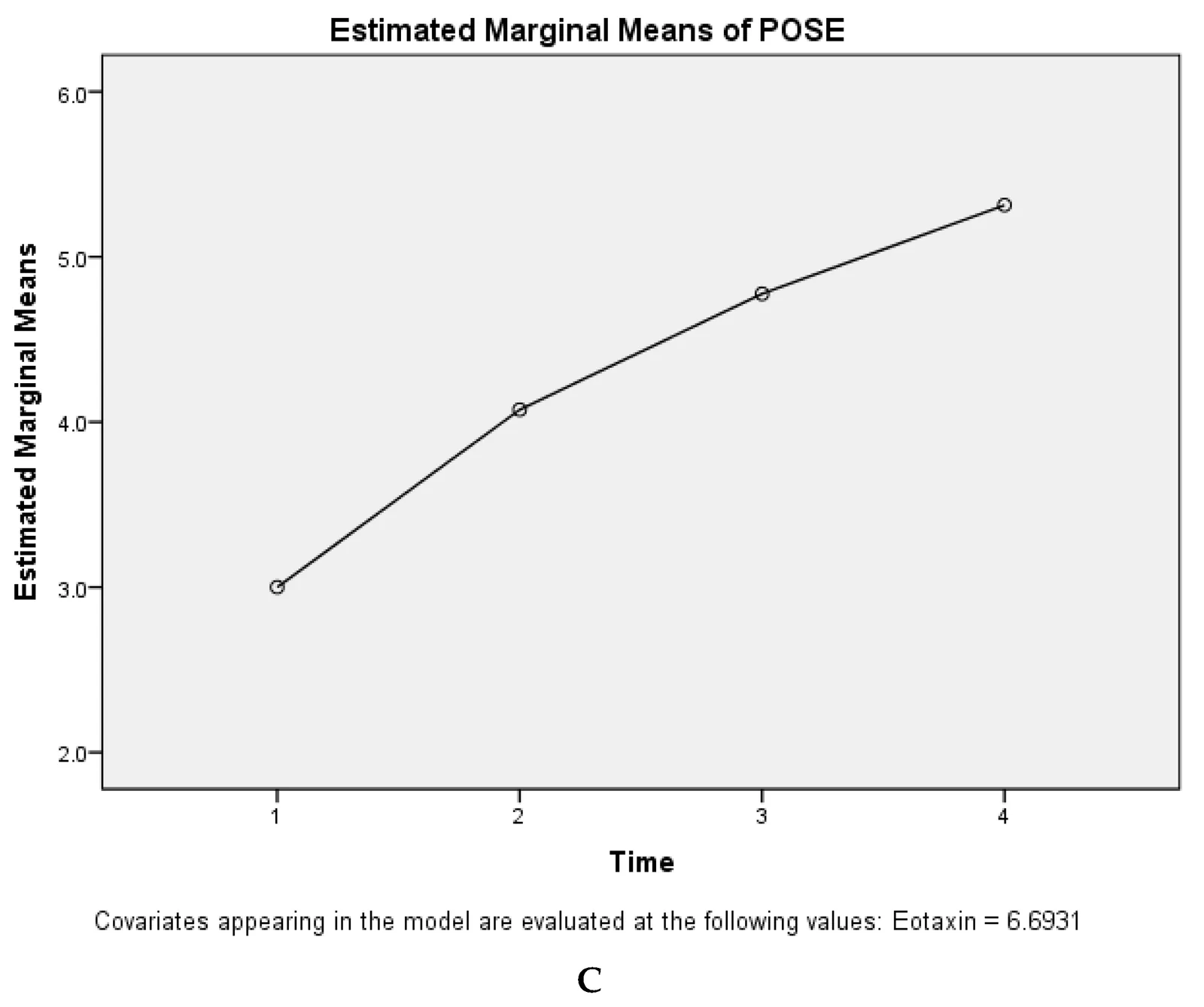
Longitudinal evolution of POSE scores according to periostin (**A**), procalcitonin (**B**), eotaxin (**C**) levels and recurrence status over 3 years. Time represents the postoperative follow-up time: 1 = immediately after surgery; 2 = 6 months; 3 = 1 year; 4 = 3 years.

Overall, the 3-year MANCOVA models demonstrated significant longitudinal associations between periostin, eotaxin, and procalcitonin and postoperative inflammatory burden, as reflected by POSE scores over time. Among the evaluated biomarkers, eotaxin showed the strongest multivariate association with longitudinal POSE scores, followed by periostin and procalcitonin, all exhibiting large effect sizes (Table 8). As illustrated in Figure 3, the POSE scores increased progressively throughout the follow-up period, with similar temporal trajectories observed for all three biomarkers.

## 4. Discussion

CRSwNP is a heterogeneous disease, so current ESS could markedly improve the clinical symptoms of patients, but remain a large proportion of patients who suffer a relapse. Therefore, an early and appropriate method to predict the prognosis and recurrence of CRSwNP are extremely important. The present study demonstrates that several circulating markers are significantly associated with disease severity, postoperative inflammatory burden, and recurrence risk in patients with CRSwNP.

This study identified peripheral blood eosinophilia and asthma as the strongest baseline predictors of postoperative recurrence in CRSwNP, whereas conventional clinical parameters, including preoperative SNOT-22, Lund–Mackay, and endoscopic scores, did not differ significantly between recurrence groups. These findings suggest that the underlying type 2 inflammatory endotype is a stronger determinant of long-term outcomes than baseline clinical severity. Consistent with our results, Wang et al. demonstrated that the combination of blood eosinophil percentage and asthma history significantly improves recurrence prediction, while Brown et al. further emphasized that inflammatory endotyping, particularly eosinophilia and asthma, better reflects disease severity than conventional clinical classification alone [21,22]. Peripheral blood eosinophilia emerged as the strongest clinical predictor of postoperative recurrence. These finding is consistent with previous studies identifying eosinophilic inflammation as the principal driver of disease persistence and postoperative relapse in CRSwNP. Kim et al. confirmed, in a meta-analysis of over 6000 patients, that eosinophilic CRSwNP is characterized by higher blood eosinophil levels, greater disease severity, and increased postoperative recurrence [23].

Importantly, although peripheral eosinophilia remains the reference clinical predictor, the investigated serum biomarkers provide complementary information regarding inflammatory activity and tissue remodeling. Biomarkers such as periostin, eotaxin, IL-33 and procalcitonin may therefore contribute to a more comprehensive biological characterization of patients and improve postoperative risk stratification when interpreted alongside established clinical predictors.

Before surgical treatment, baseline serum periostin, eotaxin, and procalcitonin were associated with objective measures of disease severity, including radiological and endoscopic scores, whereas no significant associations were observed with baseline symptom burden as assessed by SNOT-22. This discrepancy is consistent with previous evidence indicating that patient-reported symptom severity does not necessarily correspond to the extent of sinonasal inflammation or radiological disease burden in chronic rhinosinusitis [20].

Interestingly, stronger correlations were observed for postoperative than for preoperative outcomes. Among the evaluated biomarkers, periostin, eotaxin, and procalcitonin consistently showed strong relationships with both subjective and objective measures of disease activity, whereas IL-33 displayed limited predictive value throughout follow-up. Moderate correlations were observed between these biomarkers and SNOT-22 scores, whereas substantially stronger correlations were identified with POSE scores at both 1- and 3-year follow-up intervals. The observed correlations suggest that periostin, eotaxin, and procalcitonin are associated with radiological disease burden in CRS, with stronger associations observed for postoperative than preoperative Lund–Mackay scores. Conversely, serum IL-33 did not demonstrate a significant relationship with radiological severity, indicating limited utility as a systemic biomarker of disease extent in this cohort. These findings suggest that circulating biomarkers may better reflect persistent mucosal inflammatory activity and tissue remodeling than symptom perception alone.

Eotaxin demonstrated one of the strongest correlations with POSE scores and emerged as an significant predictor of recurrence in multivariate Cox analysis. These findings are consistent with the study by Wang et al., who identified serum eotaxin as a significant predictor of postoperative recurrence in CRSwNP patients based on multiplex cytokine profiling [24]. Eotaxin plays a critical role in eosinophil recruitment and activation, central mechanisms in type 2 inflammatory CRSwNP. The persistence of elevated eotaxin levels in recurrent patients from our cohort supports the hypothesis that sustained eosinophilic inflammation contributes to postoperative relapse and progressive mucosal disease.

The observed association between periostin and disease severity aligns with previous studies identifying periostin as a key biomarker of Th2-driven inflammation and tissue remodeling in CRSwNP. Ozturk Yilmaz et al. reported significant correlations between periostin expression, tissue eosinophilia, and postoperative outcomes, suggesting a prognostic role for periostin in disease recurrence and progression [25]. Our results further extend these observations by demonstrating that elevated periostin levels are associated not only with recurrence but also with longitudinal worsening of POSE scores over a 3-year follow-up period. Given the known role of periostin in extracellular matrix remodeling and eosinophilic activation, these findings reinforce its importance as a marker of persistent inflammatory activity.

Ninomiya et al. found a positive association between serum periostin and tissue eosinophil infiltration in CRSwNP patients [26], while Kim et al. reported higher periostin mRNA expression and protein levels in eosinophilic compared with non-eosinophilic nasal polyps [27]. Similarly, Qin et al. demonstrated significant correlations between serum periostin, tissue periostin expression, and peripheral blood eosinophil percentage [28]. These findings are consistent with our results, as patients who developed recurrence exhibited significantly higher baseline serum periostin levels, supporting the association between periostin and type 2 eosinophilic inflammation. Several studies have also demonstrated significant correlations between periostin (serum or tissue) and radiological disease severity assessed by the Lund–Mackay score, while Mueller et al. reported that increasing periostin concentrations in nasal mucus were associated with worsening SNOT-22 scores during follow-up [29]. In contrast, preoperative SNOT-22 and Lund–Mackay scores were not significantly different between recurrence groups in our cohort, suggesting that circulating periostin may provide additional prognostic information beyond conventional clinical and radiological assessment.

Periostin expression rises during active CRS and falls after effective medical or surgical therapy, suggesting its potential usefulness for monitoring treatment response [30]. Similar to findings in asthma, periostin levels in CRS are linked to prognosis and therapeutic outcome. Evaluating recurrence 28 days after ESS, Zhang et al. reported that periostin levels in epithelial brushings from the frontal recess decreased by 3 months postoperatively, becoming similar to controls [31]. Conversely, Kanemitsu et al. found that serum periostin did not decrease at 12 months in seven patients who experienced recurrence after ESS, suggesting serum periostin changes may reflect NP recurrence [32]. In a larger study of 338 CRS patients, 278 with CRSwNP, Oka et al. proposed serum IgG4 and periostin as potential biomarkers for predicting postoperative recurrence [33]. Wei et al. likewise reported a significant association between tissue periostin and polyp recurrence after ESS [34]. The influence of bronchial asthma on periostin levels remains controversial, largely because previous studies did not account for asthma phenotypes and CRSwNP endotypes. In stable asthma, serum periostin has been reported to be significantly elevated in eosinophilic patients with severe, adult-onset disease and worse lung function [32,34]. Likewise, patients with eosinophilic CRSwNP and asthma show higher serum periostin than non-eosinophilic CRSwNP patients or healthy controls. Similarly, asthma was significantly more prevalent among patients with recurrence in our cohort, suggesting that periostin may reflect the persistent type 2 inflammatory milieu characteristic of this high-risk subgroup. Further research that stratifies by asthma and CRSwNP endotypes is needed to clarify whether asthma is a meaningful confounder of periostin levels in CRSwNP.

Although peripheral blood eosinophilia remained the strongest clinical predictor of recurrence, procalcitonin demonstrated the highest discriminative ability among the investigated circulating biomarkers, achieving the largest AUC. While traditionally associated with bacterial inflammatory responses, procalcitonin has increasingly been implicated in chronic inflammatory conditions. Bilici et al. previously demonstrated significantly elevated serum and tissue procalcitonin levels in CRSwNP patients compared with controls, suggesting a role in nasal polyp pathogenesis [35]. Our results extend these findings by demonstrating its strong association with recurrence risk and longitudinal postoperative inflammatory severity. Elevated procalcitonin levels may therefore reflect sustained inflammatory activation and epithelial dysfunction contributing to disease persistence.

In contrast, serum IL-33 did not correlate significantly with Lund–Mackey, SNOT-22 or POSE scores and failed to predict recurrence within our cohort. These findings differ partially from previous studies that suggested a role for IL-33 in CRSwNP severity and eosinophilic inflammation. Zielińska-Bliźniewska et al. reported elevated serum IL-33 levels associated with eosinophilic CRSwNP severity [12], while Zhang et al. demonstrated that serum IL-33 could predict postoperative recurrence and distinguish CRSwNP endotypes [36]. Conversely, Ozturan et al. found no significant differences in serum IL-33 levels between CRSwNP and control groups, suggesting inconsistent systemic expression patterns [37]. Our findings appear to support the latter observations and may indicate that circulating IL-33 does not consistently reflect chronic postoperative inflammatory activity. Although tissue IL-33 did not demonstrate significant systemic correlations in our cohort, in a previous study, Porfire et al. demonstrated significant correlations between tissue IL-33 expression, osteitis severity, eosinophilia, and postoperative disease burden in CRSwNP [38]. This discrepancy across studies could be explained by differences in patient endotypes, eosinophilic burden, disease stage, sample timing, or local tissue-specific IL-33 expression rather than systemic release.

The dual function—linking extracellular matrix remodeling with immune activation—provides a plausible explanation for the strong associations observed in our study between periostin, eotaxin, procalcitonin and miR-125b. Multiple studies have discovered an overexpression of miR-125b in nasal polyps [18], increased serum levels of miR-125b were also found in asthma patients [39], which proved to have potential involvement in the EMT process related to the etiopathology of asthma. These findings suggest that although tissue microRNAs may participate in local inflammatory regulation, circulating biomarkers may provide superior predictive utility for postoperative recurrence.

The univariate analysis further demonstrated that patients with recurrence exhibited significantly higher serum periostin, eotaxin and procalcitonin levels compared with controlled patients. These findings support the hypothesis that persistent systemic inflammatory activation and remodeling processes contribute to postoperative relapse.

Time-dependent ROC analysis demonstrated that predictive performance generally declined over time, although several biomarkers remained clinically relevant at 3 years, particularly procalcitonin, periostin, and eotaxin. This pattern likely reflects the increasing influence of additional environmental, immunological, and therapeutic factors during long-term disease evolution. Nevertheless, the persistence of acceptable predictive performance at extended follow-up intervals supports the utility of these biomarkers for medium-term prognostic stratification. These results are supported by previous studies showing the power of these biomarkers in predicting CRSwNP prognosis [12,23,34,40].

The Kruskal–Wallis test and MANCOVA demonstrated that periostin, eotaxin, and procalcitonin were significantly associated with POSE evolution over the 3-year follow-up period. Among the evaluated biomarkers, eotaxin exhibited the strongest multivariate effect, followed by periostin and procalcitonin, indicating that these biomarkers are closely related to the long-term evolution of endoscopic disease activity rather than to isolated postoperative findings. These findings indicate that these biomarkers may not only predict recurrence events but may also reflect the overall intensity and persistence of mucosal inflammation during follow-up. This brings new proves to the study conducted by Guo et.al and emphasizes the aim of identifying noninvasive biomarkers associated with difficult-to-treat CRS, even before surgery [41].

Beyond disease-specific biomarkers, growing evidence supports the clinical utility of readily available systemic inflammatory markers for disease characterization, prognostic assessment, and treatment monitoring in chronic inflammatory disorders. Hematological inflammatory indices, including the neutrophil-to-lymphocyte ratio (NLR), systemic immune-inflammation index (SII), and pan-immune-inflammation value (PIV), as well as circulating biomarkers such as C-reactive protein (CRP) and fibrinogen, have demonstrated promising diagnostic and prognostic performance by reflecting the underlying systemic inflammatory burden [42,43]. These findings reinforce the concept that systemic immune-inflammatory profiling may complement disease-specific circulating biomarkers, supporting more accurate risk stratification and personalized management. Within this context, our findings further support the potential role of circulating periostin, eotaxin, and procalcitonin as accessible biomarkers for identifying CRSwNP patients at increased risk of postoperative recurrence.

Current literature increasingly supports multidimensional approaches for postoperative follow-up. Joustra et al. emphasized that long-term disease control remains difficult to achieve even years after surgery, with nearly half of CRS patients remaining uncontrolled according to EPOS criteria [44]. Similarly, Cotter et al. proposed the Chronic Rhinosinusitis Control Test (CRCT) as a multidimensional patient-reported instrument for longitudinal disease assessment [45]. Our results complement these approaches by demonstrating that objective biomarker profiling may provide additional prognostic information beyond symptom-based classifications alone. These findings are consistent with the review by Caminati et al., who proposed that remission in CRSwNP should include not only symptom control, but also objective assessment of inflammatory activity and disease severity [46]. Similarly, our study demonstrated that periostin, eotaxin and procalcitonin were associated with recurrence risk and persistent postoperative inflammatory burden. While Caminati et al. emphasized the lack of validated biomarkers for defining remission, our results suggest that circulating biomarkers may complement clinical and endoscopic evaluation and contribute to more precise long-term monitoring of CRSwNP patients.

This study has several limitations. First, the relatively small sample size, limited number of recurrence events, and single-center design may restrict the generalizability of the findings. Consequently, the multivariable models and ROC-derived cut-off values should be considered exploratory and may be affected by overfitting. In addition, internal validation procedures, such as bootstrapping or cross-validation, were not performed. Therefore, the reported predictive performance and cut-off values require external validation in larger, independent, preferably multicenter cohorts before routine clinical implementation.

Second, although tissue eosinophilia is a well-established predictor of CRSwNP recurrence, it was not included in the predictive model because its routine clinical application remains limited by the lack of standardized cut-off values and considerable inter-observer variability in histopathological assessment. Therefore, the present study focused on standardized, readily available preoperative clinical, laboratory, and imaging parameters that can be easily implemented in routine clinical practice.

In addition, although the quantitative peripheral blood eosinophil percentage demonstrated excellent predictive performance, the binary classification of eosinophilia represents a simplified variable that may reduce statistical precision, particularly in relatively small cohorts. Furthermore, although peripheral blood eosinophilia remained the strongest clinical predictor of recurrence, the present study was not designed to determine whether the investigated circulating biomarkers provide incremental predictive value beyond eosinophilia. Biomarker levels were assessed only at baseline, and serial postoperative measurements could provide additional information regarding disease progression and long-term remission.

Furthermore, serum IL-33 did not demonstrate significant prognostic value in our cohort, which may reflect differences in disease endotypes, sample size, or the predominantly local tissue-specific activity of this cytokine rather than its systemic expression.

## 5. Conclusions

The investigated circulating biomarkers, particularly periostin, eotaxin, and procalcitonin, showed promising prognostic potential for postoperative recurrence in CRSwNP. Future prospective multicenter studies with larger cohorts are warranted to validate their prognostic value and define their role in personalized postoperative management.

## Figures and Tables

**Table 1:** Baseline characteristics of patient cohort according recurrence status of CRSwNP.

Baseline Characteristics	Controlled and Partial Controlled CRSwNP	Recurrent CRSwNP	*p*-Value
Total number of patients, n	49	20	
Age mean (years) ± SD	48.71 ± 11.76	47.25 ± 10.9	0.633
Males n (%)	34 (69.4%)	13 (65%)	0.723
Females n (%)	15 (30.6%)	7 (35%)	
Asthma (Yes) n (%)	8 (16.3%)	17 (85%)	<0.001
NSAID intolerance (Yes) n (%)	9 (18.4%)	2 (10%)	0.272
Environmental allergies (Yes) n (%)	7 (14.3%)	6 (30%)	0.389
Smokers (Yes) n (%)	12 (24.5%)	5 (25%)	0.964
Eosinophilia (Yes) n (%)	8 (16.3%)	20 (100%)	<0.001
Preoperatory SNOT-22 mean ± SD	42.92 ± 18.56	50.05 ± 21.82	0.174
Preoperatory Lund–Mackay score median (IQR)	7.5 (3)	9 (3.5)	0.064
Preoperatory endoscopic score median (IQR)	2 (1)	2.5 (1)	0.064
Baseline Periostin median (IQR)	287.44 (40.64)	321.22 (13.45)	<0.001
Baseline Eotaxin median (IQR)	5.57 (1.7)	9.48 (2.97)	<0.001
Baseline IL-33 median (IQR)	74 (102.06)	50.39 (165.61)	0.802
Baseline Procalcitonin mean ± SD	58.54 ± 6.35	68.05 ± 6.48	<0.001

**Table 2:** Associations between baseline serum biomarker levels and preoperative disease severity.

	Periostin	Eotaxin	IL-33	Procalcitonin
Lund–Mackay score	0.256 (0.034)	0.337 (0.005)	0.188 (0.173)	0.327 (0.006)
SNOT-22 score	0.124 (0.299)	0.201 (0.091)	0.103 (0.173)	0.147 (0.218)
Endoscopic score	0.275 (0.022)	0.278 (0.021)	0.151 (0.275)	0.385 (0.001)
*N*	69	69	69	69

Values are presented as Spearman’s rank correlation coefficients, with *p*-values in parentheses.

**Table 3:** Univariate correlations between serum biomarkers and postoperative outcomes (SNOT-22 and POSE scores at 1 year and 3 years follow-up)—Spearman’s Correlation Coefficient, *p* < 0.05.

	Periostin	Eotaxin	IL-33	Procalcitonin
Post-operatory Lund–Mackay score	0.492 (0.001)	0.521 (0.001)	−0.079 (0.644)	0.576 (0.001)
SNOT-22 score at 1 year	0.309 (0.010)	0.393 (0.001)	−0.129 (0.353)	0.338 (0.004)
POSE score 1 year	0.720 (0.001)	0.853 (0.001)	0.014 (0.919)	0.789 (0.001)
SNOT-22 score 3 years	0.288 (0.016)	0.351 (0.003)	−0.034 (0.806)	0.292 (0.015)
POSE score 3 years	0.591 (0.001)	0.645 (0.001)	−0.003 (0.982)	0.586 (0.001)
*N*	69	69	69	69

Values are presented as Spearman’s rank correlation coefficients, with *p*-values in parentheses.

**Table 4:** Spearman’s correlations between serum biomarkers and miRs.

	miR 125b r (*p*-Value)	miR 203a r (*p*-Value)
Periostin	0.271 (0.025)	0.202 (0.095)
Eotaxin	0.342 (0.004)	0.200 (0.099)
Procalcitonin	0.296 (0.014)	0.194 (0.110)
Serum IL-33	−0.232 (0.092)	−0.097 (0.486)
*N*	69	69

Values are presented as Spearman’s correlation coefficients (*r*), with *p*-values shown in parentheses.

**Table 5:** Univariate analysis of serum biomarkers associated with nasal polyposis recurrence.

Biomarker	Controlled (Median)	Relapse (Median)	Mann–Whitney U	*p*-Value
Periostin (ng/mL)	287.44	321.22	200.5	<0.001
Eotaxin (pg/mL)	5.57	9.48	207.0	<0.001
Procalcitonin (pg/mL)	57.66	68.05	191.5	<0.001
IL-33 serum (pg/mL)	74.00	50.39	279.5	0.802

**Table 6:** Univariate Cox proportional hazards regression model for recurrence prediction.

Biological Variable	HR	95% CI	*p*-Value
Periostin	1.033	1.007–1.060	0.010
Eotaxin	1.554	1.258–1.919	<0.001
Procalcitonin	1.183	1.085–1.290	<0.001
Serum IL-33	1.000	0.996–1.004	0.835

**Table 7:** ROC analysis of peripheral blood eosinophilia and circulating biomarkers for predicting postoperative recurrence.

Variables	AUC (95% CI)	Cut-Off Value	Sensitivity	Specificity	*p*-Value
Peripheral blood eosinophilia	0.959 (0.919–1.000)	5.0%	100%	81.6%	<0.001
Periostin	0.758 (0.605–0.912)	300.1 ng/mL	77.8%	64.7%	0.001
Eotaxin	0.757 (0.607–0.907)	6.9 pg/mL	77.8%	62.7%	0.001
Procalcitonin	0.805 (0.662–0.947)	60.3 pg/mL	80.0%	69.5%	<0.001
Serum IL-33	0.552 (0.402–0.702)	74.8 pg/mL	50.0%	52.0%	0.346

**Table 8:** Repeated-measures MANCOVA showing the association between biomarkers and POSE scores.

Biomarker	Wilks’ Λ	F (df1, df2)	*p*-Value	Partial η^2^
Periostin	0.309	F(6,16) = 5.964	0.002	0.691
Eotaxin	0.128	F(6,16) = 18.180	<0.001	0.872
Procalcitonin	0.408	F(5,17) = 4.938	0.006	0.592

## Data Availability

No data available due to privacy concerns.

## References

[1] Albu S. (2020). Chronic Rhinosinusitis-An Update on Epidemiology, Pathogenesis and Management. J. Clin. Med..

[2] Fokkens W.J., Lund V.J., Hopkins C., Hellings P.W., Kern R., Reitsma S., Toppila-Salmi S., Bernal-Sprekelsen M., Mullol J., Alobid I. (2020). European Position Paper on Rhinosinusitis and Nasal Polyps 2020. Rhinology.

[3] Davies C., Wu F., Huang E.Y., Takashima M., Rowan N.R., Ahmed O.G. (2023). Central Compartment Atopic Disease as a Pathophysiologically Distinct Subtype of Chronic Rhinosinusitis: A Scoping Review. Sinusitis.

[4] Jutel M., Agache I., Zemelka-Wiacek M., Akdis M., Chivato T., Del Giacco S., Gajdanowicz P., Gracia I.E., Klimek L., Lauerma A. (2023). Nomenclature of allergic diseases and hypersensitivity reactions: Adapted to modern needs: An EAACI position paper. Allergy.

[5] Bachert C., Marple B., Schlosser R.J., Hopkins C., Schleimer R.P., Lambrecht B.N., Bröker B.M., Laidlaw T., Song W.-J. (2020). Adult chronic rhinosinusitis. Nat. Rev. Dis. Prim..

[6] Yao Y., Yang C., Yi X., Xie S., Sun H. (2020). Comparative analysis of inflammatory signature profiles in eosinophilic and noneosinophilic chronic rhinosinusitis with nasal polyposis. Biosci. Rep..

[7] Ebenezer J.A., Christensen J.M., Oliver B.G., Oliver R.A., Tjin G., Ho J., Habib A.R., Rimmer J., Sacks R., Harvey R.J. (2017). Periostin as a marker of mucosal remodelling in chronic rhinosinusitis. Rhinology.

[8] Gilbert D.N. (2011). Procalcitonin as a biomarker in respiratory tract infection. Clin. Infect. Dis..

[9] Coyle A.J., Lloyd C., Tian J., Nguyen T., Erikkson C., Wang L., Ottoson P., Persson P., Delaney T., Lehar S. (1999). Crucial role of the interleukin 1 receptor family member T1/ST2 in T helper cell type 2-mediated lung mucosal immune responses. J. Exp. Med..

[10] Lam E.P., Kariyawasam H.H., Rana B.M., Durham S.R., McKenzie A.N., Powell N., Orban N., Lennartz-Walker M., Hopkins C., Ying S. (2016). IL-25/IL-33-responsive TH2 cells characterize nasal polyps with a default TH17 signature in nasal mucosa. J. Allergy Clin. Immunol..

[11] Kim D.K., Jin H.R., Eun K.M., Mo J.H., Cho S.H., Oh S., Cho D., Kim D.W. (2017). The role of interleukin-33 in chronic rhinosinusitis. Thorax.

[12] Zielińska-Bliźniewska H., Paprocka-Zjawiona M., Merecz-Sadowska A., Zajdel R., Bliźniewska-Kowalska K., Malinowska K. (2022). Serum IL-5, POSTN and IL-33 levels in chronic rhinosinusitis with nasal polyposis correlate with clinical severity. BMC Immunol..

[13] Yao T., Kojima Y., Koyanagi A., Yokoi H., Saito T., Kawano K., Furukawa M., Kusunoki T., Ikeda K. (2009). Eotaxin-1, -2, and -3 immunoreactivity and protein concentration in the nasal polyps of eosinophilic chronic rhinosinusitis patients. Laryngoscope.

[14] Yamada T., Miyabe Y., Ueki S., Fujieda S., Tokunaga T., Sakashita M., Kato Y., Ninomiya T., Kawasaki Y., Suzuki S. (2019). Eotaxin-3 as a plasma biomarker for mucosal eosinophil infiltration in chronic rhinosinusitis. Front. Immunol..

[15] De Corso E., Settimi S., Montuori C., Cantiani A., Corbò M., Di Bella G.A., Sovardi F., Pagella F., Rigante M., Passali G.C. (2023). How to manage recurrences after surgery in CRSwNP patients in the biologic era: A narrative review. Acta Otorhinolaryngol. Ital..

[16] Lund V.J., Mackay I.S. (1993). Staging in Rhinosinusitus. Rhinology.

[17] Lildholdt T., Rundcrantz H., Lindqvist N. (1995). Efficacy of Topical Corticosteroid Powder for Nasal Polyps: A Double-Blind, Placebo-Controlled Study of Budesonide. Clin. Otolaryngol. Allied Sci..

[18] Gata A., Leucuta D.C., Budisan L., Raduly L., Trombitas V.E., Berindan-Neagoe I., Albu S. (2024). MicroRNA-125b Is a Potential Predictor of Surgical Outcomes in Chronic Rhinosinusitis with Nasal Polyps. Am. J. Rhinol. Allergy.

[19] Wright E.D., Agrawal S. (2007). Impact of Perioperative Systemic Steroids on Surgical Outcomes in Patients With Chronic Rhinosinusitis With Polyposis: Evaluation With the Novel Perioperative Sinus Endoscopy (POSE) Scoring System. Laryngoscope.

[20] Gata A., Raduly L., Budișan L., Bajcsi A., Ursu T.M., Chira C., Dioșan L., Berindan-Neagoe I., Albu S. (2024). Machine Learning Model Predicts Postoperative Outcomes in Chronic Rhinosinusitis with Nasal Polyps. Clin. Otolaryngol..

[21] Wang X., Meng Y., Lou H., Wang K., Wang C., Zhang L. (2021). Blood eosinophil count combined with asthma history could predict chronic rhinosinusitis with nasal polyp recurrence. Acta Otolaryngol..

[22] Brown H.J., Tajudeen B.A., Kuhar H.N., Gattuso P., Batra P.S., Mahdavinia M. (2021). Defining the Allergic Endotype of Chronic Rhinosinusitis by Structured Histopathology and Clinical Variables. J. Allergy Clin. Immunol. Pract..

[23] Kim D.H., Kim S.W., Basurrah M.A., Hwang S.H. (2022). Clinical and Laboratory Features of Various Criteria of Eosinophilic Chronic Rhinosinusitis: A Systematic Review and Meta-Analysis. Clin. Exp. Otorhinolaryngol..

[24] Wang G., Zheng H., Chen X., Zheng J., Zhan J., Li R., Qi Y., Ye Y., Zeng M., Wei X. (2022). Exploration of Predictive Biomarkers for Postoperative Recurrence in Chronic Rhinosinusitis with Nasal Polyps Based on Serum Multiple-Cytokine Profiling. Mediat. Inflamm..

[25] Yilmaz G.O., Cetinkaya E.A., Eyigor H., Ellidag H.Y., Balaban K., Selcuk O.T., Yilmaz G., Gur O.E. (2022). The diagnostic importance of periostin as a biomarker in chronic rhinosinusitis with nasal polyp. Eur. Arch. Otorhinolaryngol..

[26] Ninomiya T., Noguchi E., Haruna T., Hasegawa M., Yoshida T., Yamashita Y. (2018). Periostin as a novel biomarker for postoperative recurrence of chronic rhinosinitis with nasal polyps. Sci. Rep..

[27] Kim D.W., Kulka M., Jo A., Eun K.M., Arizmendi N., Tancowny B.P. (2017). Cross-talk between human mast cells and epithelial cells by IgE-mediated periostin production in eosinophilic nasal polyps. J. Allergy Clin. Immunol..

[28] Qin Z., Li X., Cai X., Li J., Zhu H., Ma Y. (2016). Periostin: A novel biomarker for chronic rhinosinusitis. B-ENT.

[29] Mueller S.K., Wendler O., Nocera A., Grundtner P., Schlegel P., Agaimy A. (2019). Escalation in mucus cystatin 2, pappalysin-A, and periostin levels over time predict need for recurrent surgery in chronic rhinosinusitis with nasal polyps. Int. Forum Allergy Rhinol..

[30] Lehmann A.E., Scangas G.A., Bergmark R.W., El Rassi E., Stankovic K.M., Metson R. (2019). Periostin and inflammatory disease: Implications for chronic rhinosinusitis. Otolaryngol. Head. Neck Surg..

[31] Zhang W., Hubin G., Endam L.M., Al-Mot S., Filali-Mouhim A., Desrosiers M. (2012). Expression of the extracellular matrix gene periostin is increased in chronic rhinosinusitis and decreases following successful endoscopic sinus surgery. Int. Forum Allergy Rhinol..

[32] Kanemitsu Y., Kurokawa R., Ono J., Fukumitsu K., Takeda N., Fukuda S. (2020). Increased serum periostin levels and eosinophils in nasal polyps are associated with the preventive effect of endoscopic sinus surgery for asthma exacerbations in chronic rhinosinusitis patients. Int. Arch. Allergy Immunol..

[33] Oka A., Ninomiya T., Fujiwara T., Takao S., Sato Y., Gion Y. (2020). Serum IgG4 as a biomarker reflecting pathophysiology and post-operative recurrence in chronic rhinosinusitis. Allergol. Int..

[34] Wei Y., Ma R., Zhang J., Wu X., Yu G., Hu X. (2018). Excessive periostin expression and Th2 response in patients with nasal polyps: Association with asthma. J. Thorac. Dis..

[35] Bilici S., Cinar Z., Yigit O., Cakir M., Yigit E., Uzun H. (2019). Does procalcitonin have a role in the pathogenesis of nasal polyp?. Eur. Arch. Otorhinolaryngol..

[36] Zhang Y., Zhu K., Chen J., Xia C., Yu C., Gao T., Yan J., Zhao B., Ren X. (2022). Predictive Values of Serum IL-33 and sST2 in Endotypes and Postoperative Recurrence of Chronic Rhinosinusitis with Nasal Polyps. Mediat. Inflamm..

[37] Ozturan A., Eyigor H., Eyigor M., Osma U., Yilmaz M.D., Selcuk O.T., Renda L., Gultekin M. (2017). The role of IL-25 and IL-33 in chronic rhinosinusitis with or without nasal polyps. Eur. Arch. Otorhinolaryngol..

[38] Porfire Irimia I.M., Berindan-Neagoe I., Budisan L., Leucuta D.C., Gata A., Minoiu A.C., Georgescu B.A., Covaliu B.F., Albu S. (2023). Tissue Interleukin-33: A Novel Potential Regulator of Innate Immunity and Biomarker of Disease Severity in Chronic Rhinosinusitis with Nasal Polyps. J. Clin. Med..

[39] Specjalski K., Jassem E. (2019). MicroRNAs: Potential Biomarkers and Targets of Therapy in Allergic Diseases?. Arch. Immunol. Ther. Exp..

[40] Seah J.J., Thong M., Wang Y. (2023). The Diagnostic and Prognostic Role of Biomarkers in Chronic Rhinosinusitis. Diagnostics.

[41] Guo C.L., Liao B., Liu J.X., Pan L., Liu Z. (2021). Predicting difficult-to-treat chronic rhinosinusitis by noninvasive biological markers. Rhinology.

[42] Hepkarşı S., Aliyeva A., Kirazlı G., Kirazlı T. (2025). Evaluating inflammatory markers in sudden sensorineural hearing loss: The neutrophil-to-lymphocyte ratio, the systemic immune inflammation index, and the pan immune inflammation value as prognostic tools. Osman. Tıp Derg..

[43] Aliyeva A., Hashimli R., Yılmaz B. (2025). Hematological Biomarkers of the Obstructive Sleep Apnea Syndrome: A Machine Learning-Based Diagnostic and Prognostic Model. J. Clin. Med..

[44] Joustra G.E., den Heijer M.C., Al Yousef R.Q.H., Vermeulen K.M., Halmos G.B., Korsten-Meijer A.G.W., Feijen R.A. (2025). Long-term clinical control in chronic rhinosinusitis: Outcomes more than five years after surgery. Eur. Arch. Otorhinolaryngol..

[45] Cotter R.A., Lee C.W., Wilson K., Althoff S.F., Alsaleh S., Anselmo-Lima W.T., Bernal-Sprekelsen M., Chandra R.K., Constantinidis J., Fokkens W.J. (2026). Development and psychometric validation of the Chronic Rhinosinusitis Control Test. Rhinology.

[46] Caminati M., De Corso E., Ottaviano G., Pipolo C., Schiappoli M., Seccia V., Spinelli F.R., Savarino E.V., Gisondi P., Senna G. (2024). Remission in Type 2 Inflammatory Diseases: Current Evidence, Unmet Needs, and Suggestions for Defining Remission in Chronic Rhinosinusitis with Nasal Polyps. Curr. Allergy Asthma Rep..

